# The Study of Epidemic Diseases; Illustrated by the Pestilences of London

**Published:** 1858-01

**Authors:** E. Headlam Greenhow


### ON THE STUDY OF EPIDEMIC DISEASES: ILLUSTRATED BY THE PESTILENCES OF LONDON.

By E. HEADLAM GREENHOW, M.D.

(*Read November* 2, 1857.)

VISITATIONS of epidemic sickness have, perhaps, rarely failed to attract public attention. They have not unfrequently excited public alarm. If, on the one hand, they have often called forth the active practice of some of the noblest virtues of the human character—brotherly love, self-denial, charity and courage, they have, alas! too often, on the other hand, been the means of temporarily slackening the bonds of social life, and of letting loose some of the worst and most depraved passions of human nature. The history of epidemic diseases forms one of the most interesting subjects of medical inquiry. The investigation of the causes which produce, and of the laws that govern, epidemic visitations, is one of the most important subjects to which the attention of the physician can be directed. Whilst other diseases are frequently attributable to accident, to personal habits, or to individual constitution, epidemic diseases are evidently produced by causes of a more extensive kind, which act on mankind in the aggregate. However influenced in their course or their result by the condition of individuals, the liability to their invasion is shared at once by large sections of the community; and if they are to be averted, this can only be effected by measures that are co-extensive with this tendency.

The study of epidemic diseases has engaged the attention of many of the ablest members of the medical profession from the days of Hippocrates down to our own time. The treatises on epidemics of the physician of Cos contain matter well worthy of careful consideration by ourselves. The most practical physicians have, indeed, ever been the most successful and the most philosophical inquirers into the causes and relations of epidemics. But that I shall hereafter have occasion to refer to his writings, it would scarcely be needful to remind you of the admirable treatises on the epidemics of the seventeenth century, by our own distinguished Sydenham, or of the works of his equally eminent contemporaries, Willis and Morton, who also flourished towards the close of that great epidemic period. The writings of these bright lights of the medical profession abound with important facts and observations of undying interest, because their knowledge was the result of practical experience and personal observation carefully noted and honestly recorded. However much science may advance or theory may change, such information never becomes obsolete, and the writings in which it is contained can never be carefully studied without benefit. More recently we have had many contributions to epidemiological science. Among these is the learned and graphic historical sketch of middle-age epidemics by Hecker, for the English version of which our medical literature is so deeply indebted to the learned President of this Society. The existence of this Society, devoted exclusively to the study and elucidation of epidemic diseases, attests that the interest which has heretofore attached to them is by no means on the wane. Epidemiological science has, notwithstanding, made but slow progress. In some respects, we have, perhaps, fallen somewhat behind our predecessors. In but few have we advanced materially beyond them in our knowledge of epidemic diseases, or in the philosophical investigation of their history. This has no doubt arisen in part from the long and happy immunity from pestilences enjoyed by our country after the close of the seventeenth century. It has, however, been also perhaps partly caused by the comprehensive character of the study, so opposed to the specialising system of the day. For the division of labour, which forms so important a feature of modern civilisation, seems to have been imported into medical science. Whether it be the manufacture of a needle, a scientific investigation, or the practice of medicine, the same tendency to the extreme subdivision of labour is now manifested. Each man has his speciality, to which he devotes his chief, too often his exclusive attention.

This adoption by medical men of the mechanical system of subdivided labour has in nothing proved more detrimental to the interests of science than in the study of epidemics, for the correct understanding of which a wide and enlarged grasp of the subject is indispensable. The collection of detailed facts and minute observations is, indeed, as essential a step to a just estimate of epidemic diseases as of any other subject of investigation. But observation must extend to a wider field, both as regards time, space and subject, than has been very usual, if it be designed to aid in the elucidation of a class of diseases whose history and course are so mysterious; which appear so capricious alike in the time, places and persons they attack; which so often visit us, to use the quaint language of an old writer, like "a blast from the stars," and having run their course, again leave us, without apparent reason, for an indefinite period.

It is but an imperfect knowledge of any epidemic disease that can be acquired from its study, however careful, in a single visitation, or even in a succession of visitations, in the same place. Perhaps it might even be justly affirmed that the study of a single form of epidemic disease, however extensive, or however careful, would leave many points obscure which might be rendered clear by its comparison with other diseases of the same class. Although epidemic diseases commonly seem to commence suddenly and mostly run their course in a particular place, within a comparatively short period, it is important to trace them from their origin, and to follow them to their close;—to observe their antecedents, and to note whether the public health at once returns to its pristine condition after their decline. Hence it is not sufficient to study the history, or the accompanying phenomena, of epidemic visitations. The prevailing types of disease, and the condition of the public health, both anterior and subsequent to such visitations, if carefully studied, will often throw important light upon their history and causes. Neither, in studying diseases which so obviously depend upon, or at least are largely influenced by, causes that act simultaneously upon great numbers of persons, must the attention be confined exclusively to the disease itself. Many cosmical phenomena, the state of the weather, peculiarities of season, and other evident circumstances, will deserve notice. Hence, also, it will be both interesting and useful to inquire into the influence, if any, which these several circumstances have produced upon the lower forms of organised beings, and particularly upon domestic animals and cultivated plants. Thus the careful and extended study of epidemic diseases involves the investigation of many and various circumstances anterior, concurrent, and subsequent to the visitation, whose history and causes it is desired to elucidate.

The form and character of epidemic diseases have varied greatly from period to period. The black death, the sweating sickness, and the glandular plague, which prevailed among our ancestors, have long since disappeared. He would be indeed a rash man who should dare to assert that these diseases are practically unknown amongst ourselves in the present day, because they have not been imported by human means from abroad. If the comparatively scanty commerce, and the limited international intercourse of the fourteenth and seventeenth centuries, were sufficient to introduce such fatal diseases as the black death and the plague—the first of which still prevails in certain parts of Hindostan under the name of the *Pali* plague; whilst the other is often prevalent either in Turkey, Egypt, or the adjoining countries—surely these diseases must have found their way hither by means of the more intimate intercourse of nations in the nineteenth century, notwithstanding the utmost vigilance of the quarantine department. As will presently appear, these diseases, whether they be of indigenous or of foreign origin, require a predisposition of persons or of places for their development. The *crasis* of the people, the conditions amidst which they exist, and the variations in these, coincident with changes of time, circumstance and civilisation, therefore demand the attention of the physician who has set himself the task of investigating epidemic diseases. Besides the study of these positive phenomena, important knowledge may likewise be derived from the investigation of certain negative facts. The singular immunity enjoyed at periods of almost universal epidemic visitation by the inhabitants of particular districts, by certain races, or by particular classes of persons, well deserve consideration. The Jews have often been believed to escape diseases from which the nations among whom they live as strangers have suffered severely. Although the assertion is certainly not true in respect of cholera, it is probably not without foundation in the case of some other pestilences. The sweating sickness is alleged by Caius to have confined itself almost exclusively to the upper and wealthier classes of society, and so exclusively in some of its visitations to the English race, that no aliens were attacked by it in England, and none but the English suffered from it abroad.

If the view of the subject I have here ventured to express be correct—and I express it with great deference and humility in the presence of a society devoted to the investigation of epidemic diseases—how wide the field of labour upon which the Epidemiological Society of London has entered! how plentiful the harvest it may hope to reap! When the Council of this Society did me the honour of inviting me to prepare a paper for the inauguration of the session, whose commencement dates from to-day, I thought I might, perhaps without impropriety, employ a portion of the brief time at my disposal in thus attempting to sum up shortly the most obvious heads of inquiry in the study of epidemic diseases. I have done so with much diffidence in such a presence, and rather in the expectation of furnishing a topic for profitable discussion, which may serve to elicit the opinions of my auditors, than from any hope of conveying to them any new ideas. I now propose briefly to illustrate the preceding suggestions by a reference to the epidemics of London.

By the epidemics of London I mean only the more notable diseases of this kind, and such as, if they be capable of spreading by infection, are likewise admitted by almost universal consent to spread sometimes irrespectively of personal intercourse with the infected; or to require some peculiar condition of persons or places for their full development. Thus purely contagious affections, such as small-pox and scarlatina, which, however much they may vary in *intensity* with variations of time and circumstance, vary in *amount* only in proportion to the number of persons unprotected by a previous attack or by personal idiosyncrasy, who are brought into relation with the special contagion, will not fall within the class as thus defined. The epidemics, therefore, whose history I propose to employ for my present purpose, and which might very appropriately be termed pure epidemics, will be conveniently divisible into two classes, viz.:

1. Epidemics apparently depending upon some purely atmospherical influence, which will comprise only the single form of febrile disorder, known under the name of influenza.

2. Epidemic pestilences, depending also, indeed, in a great measure, upon atmospherical influences for their production, but requiring likewise some definite condition of persons or places, peculiar to each, for their full development. Under this head, the black death, sweating sickness, plague, dysentery, or, as I propose to term it, alvine flux, and cholera (also a form of alvine flux) will be included.

The most prominent feature in the history of all these diseases is their irregularly periodical character, their tendency to recur again and again, in the epidemic form, after absences of longer or shorter duration. Such has been the history of all of them, excepting the black death, of which only one visit is recorded. Sweating sickness, plague, and cholera, have each returned several times.

Their successive appearance is next worthy of note. The black death was the distinctive pestilence of the fourteenth century. The sweating sickness occupied the last portion of the fifteenth and the first half of the sixteenth centuries. Although not exclusively, for both plague and other epidemics preceded or followed it, and occupied the intervals between the successive outbreaks, it was the distinctive pestilence of the period. It, too, passed away as the black death had previously done, and was succeeded by plague, which prevailed in an epidemic form on several occasions between the middle of the sixteenth and the end of the third quarter of the seventeenth century. As the plague period drew to a close, a pestilential form of alvine flux arose. Mixed up with the plague in 1665, this new form of epidemic committed ravages which, if small in comparison with the mortality of the great plague year, were yet such as would in our day excite the gravest alarm. This form of disease prevailed, more or less, to the close of the seventeenth century, and, although in a form less malignant and scarcely epidemic, continued during the early portion of the eighteenth century. The eighteenth century, with this exception, presents a remarkable contrast both with the preceding centuries and our own. It was free from pestilence, although not free from epidemics. There were at least ten visitations of influenza during the century, besides a much greater prevalence of fever than now. The fever death-rate, which was nearly 54 in each 10,000 persons living at the middle of the eighteenth century, has been below 39 in the middle of the present century. The general death-rate, which is now but 25, then exceeded 35 in the 1000. The number of births in London, which now very considerably exceed the deaths, were then, with very rare exceptions, exceeded by the deaths. It is, indeed, true that the records in the last and early portion of the present centuries were records not of births, but of baptisms in parish churches; but then, on the other hand, the mortuary returns were returns not of all who died, but of that portion only which were buried in parochial burial grounds. The register of baptism was then an important document that might be essential on many occasions during life. The number of the unbaptised was therefore probably more than counterbalanced by the number of persons interred in extra-parochial cemeteries. I have dwelt somewhat upon this immunity of London from pestilence during the eighteenth century, because it has always seemed to myself a fact worthy of the most careful consideration. Notwithstanding the great improvement that has taken place in the state of the public health since the middle of the eighteenth century, notwithstanding the advances that have been made during the last half century in art, science and civilisation, epidemic pestilence has re-appeared in London within the last thirty years, and on three several occasions carried off some thousands of its population within a few weeks. This pestilence, which I need scarcely mention as cholera, is of that class to which I have applied the term alvine flux; a term that seems convenient, because it comprises both the milder and the more severe forms of the epidemic.

Pestilence, on its re-appearance after the immunity of more than a century, has therefore returned to us in the same character in which it took leave of our ancestors a century and a half since, at the close of a long series of successive pestilences. It has done more—it has returned in the same form. The disease we now call cholera, and whose threatened return for a fourth time is even now occupying a share of the public attention, is identical with one of the forms of alvine flux that prevailed at the former period. Then, as at present, it was intermixed with dysentery, and probably in a larger proportion than now, because the diet and habits of the period were conducive to that form of disease. It was also confounded in name with dysentery, although its distinctive characters were duly appreciated and clearly recorded by Willis. This fact is so interesting and important, not only as a matter of history, but also from its bearing upon the origin of cholera, that I may, perhaps, be permitted to devote a brief space to its elucidation.

Willis speaks of a form of alvine flux as peculiar to London. "It hath used to reign," he says, "almost every year," and "is commonly accounted popular, and almost proper, to the place and people." "Although the word dysentery, in the common acceptation thereof, signifies a bloody flux of the belly . . . yet, saving the etymology, I shall apply that name to this *London* disease, even when it is not at all bloody. For I have often, and a great while since, observed, that there are two very different sorts of the same flux, which almost every year is wont to be so rife here about autumn, and is commonly called, in our language, *the griping of the guts;* in the one, whereof the stools were watery, and, as it were, limpid or clear, with a sudden weakening of the body; in the other, they are bloody, but tolerable." He then proceeds to give an account of them both as they were observed, and then exactly described, by him some years before, when they were rife.

"In the year 1670, about the autumnal equinox, a great many were sick of an unbloody, but a very sharp and dangerous dysentery. The distemper came upon them on a sudden, and oftentimes without any manifest cause, and reduced the patients by grievous vomiting, frequent stools, and those watery ones, in a short time to very great weakness, horrid faintings of their spirits, and destruction of their strength. I knew a great many that, though the day before they were well enough and very hearty, yet, within twelve hours, were so miserably cast down by the tyranny of this disease, that they seemed ready to expire, in that their pulse was weak and slender, a cold sweat came upon them, and their breath was short and gasping; and, indeed, many of them that wanted either fit remedies or the help of physicians, died quickly of it. This distemper raged for a whole month, but began to decrease about the middle of October and before the first of November was almost quite gone. Few at that time had bloody stools, and not many cholerick ones, but a great many had both vomitings and evacuations that were waterish, almost limpid, and in a great quantity. And whilst this common dysentery raged so severely within this city, there was scarce any one sick of it in the country, or at least above three miles off. Moreover, though very many were sick in this place, the disease did not seem to be infectious, but only to affect those that were predisposed to receive it."

After a consideration of the treatment and the causes of this London disease,—for he again affirms that it was peculiar to London, and attributes it partly, at least, to the thick smoky atmosphere of the metropolis—Willis proceeds to describe the bloody flux in terms which identify it with the dysentery of the present day. He distinguishes between the sudden onset and rapid course of the *dysenteria incruenta,* just described, and the slower progress of true dysentery, in which the patients' strength continued still so firm, that after they had been sick about a week, and gone to stool almost twenty times every day, they could rise out of their beds. "Though the bloody evacuations seemed terrible, yet the patients did not die suddenly, but continued several weeks, yea sometimes months, voiding blood every day in great quantity."* Morton also, in his *Pyretologia,*†

---

* Willis's Pharmaceutice Rationalis; translated by S. Pordage, part i, pp. 51-6. London: fol., 1684. I have preferred to quote this unexceptionable translation, to giving what might seem to be my own interpretation to the original.

† Pyretologia seu Exercitationes de Morbis Universalibus Acutis. Londini: 1692, p. 420-1.

draws a distinction between the two forms of flux that prevailed extensively in the autumn of several years following the great plague. The one form he calls dysentery, the other colliquative diarrhœa. "Civitas ferè universa hoc Morbo correpta videbatur, atque singulis septimanis tercenti, quadrigenti, quingenti plùs minus fluxu et torminibus, cæterisque dysenteriæ excruciantis, *vel* diarrhœæ colliquativæ diris symptomatis confecti."

Morton's description applies more particularly to the autumns of 1666 and 1667. Upwards of 2300 deaths from alvine plux are recorded in the bills of mortality for 1667, which, if raised in proportion to the increase of population that has since occurred, would be equal to from 11,000 to 12,000 deaths at the present time. Most of these deaths were set down in the bills as occasioned by "griping of the guts"; a much smaller number by bloody flux. To obviate errors which might easily occur at a time when the names of fatal diseases were returned by non-medical persons, I have thrown the several allied diseases of the dysenteric and diarrhœal character together, to make the class to which I have, also to avoid misconception, applied the term "alvine flux." The deaths from this class of disorders in 1670—the year referred to by Willis—amounted to upwards of 3900, which, as London now contains more than five times as many inhabitants as it then contained, was a mortality that would be represented by the deaths of nearly 20,000 persons in the present day.

It will, perhaps, be interesting if, previous to the further consideration of these several forms of pestilence, we now pause for a brief space to contrast the mortality occasioned by former pestilential visitations with that produced by the pestilential epidemic of the nineteenth century.

In his life of Edward III, Barnes* estimates the mortality occasioned in London by the visitation of black death as having amounted to 100,000 at least. Incredible as this mortality must appear, the descriptions of the extremely contagious nature of the disease and of its excessively fatal character, given both by Hecker and Barnes, as well as the panic it excited, lend countenance to the assertion. "Parents," says Barnes, "forsook their children, and wives their husbands; nor would physicians here make their visits, for neither were they able to do good to others, and they were

* The History of Edward III. By JOSHUA BARNES. Cambridge: 1688. Fol. pp. 430-33.

almost certain thereby to destroy themselves." "Thus was death without sorrow, affinity without friendship, wilful penance and dearth without scarcity, and flying without refuge or succour. For many fled from place to place because of the pestilence; some into deserts and places not inhabited, either in hope or despair: but quick-sighted destruction found them out, and nimble-footed misery was ever ready to attend them. Others, having hired boats and other vessels into which they laid up provisions, thought, or at least hoped, to elude the power of the infection; but the destroying angel, like that in the Revelations, had one foot upon the waters as well as on the land; for alas! the very air they breathed being tainted, they drew in death together with life itself."

But very scanty materials exist from which to judge of the mortality occasioned by the sweating sickness. Five visitations occurred in London, of which the first was in 1485, the last in 1551. The fourth appears to have been the most severe, and was long afterwards spoken of as the great mortality. Of the first outbreak it is alleged that scarcely one in a hundred of the sick escaped, and that many thousands were carried off during the five weeks of its continuance. Of the fifth and last visitation, it is recorded that at Shrewsbury, where it first showed itself, it was fatal to 960 persons in a few days. In London it destroyed 800 lives during the first week of the visitation. It rarely remained long in a place, and appears only to have been very violent for a period of a few days. The gross mortality it caused must, therefore, have fallen far short of that produced by plague, an intercurrent outbreak of which in 1499, is reported to have carried off 30,000 persons in London.* It is worthy of note that the fourth epidemic of sweating sickness extended to the continent, where it excited great alarm and occasioned a considerable mortality. Two thousand deaths occurred in Hamburg; eleven hundred of them in three weeks, and the chief part of them in nine days. Eight hundred persons fell victims to the pestilence in Augsburg in six days. In our own times, cholera has thrice become epidemic in this country soon after its appearance in Hamburg. The sweating sickness of the sixteenth century, after apparently originating in England, showed itself first on the continent at Hamburg. Its appearance there, however, was not until a year after its visit to England. For an accurate account of this

---

* Hecker's Epidemics of the Middle Ages, published by the Sydenham Society, p. 197, etc.

outbreak, as well as for a more detailed history of the two great pestilences of the middle ages, I beg to refer to the very interesting account of Hecker, and especially to Dr. Babington's elegant translation. It will there be seen how little reason there is for supposing the pestilence to have been imported into Hamburg from England.*

The means of estimating the ravages produced upon the population of London by the black death and the sweating sickness have thus been uncertain. Probably the calculations that have been quoted are wanting in accuracy, and can only at best be considered as approximative. Accurate and reliable data exist in the bills of mortality from which to compute the mortality occasioned by each successive outbreak of the plague, the form of pestilence which, in this long series of epidemic diseases, followed next after the sweating sickness, and, indeed, as already said, occurred intercurrently between its visitations. The London bills of mortality were first instituted on account of the plague. The number of deaths prodnced by the visitations of 1593 and subsequent plague years are therein recorded as accurately as possible, and if error exists, it lies probably rather in the under than in the over-estimate of the deaths. From these bills, and the works of Captain Graunt and Mr. John Bell, clerk to the Company of Parish Clerks, the total number of deaths from plague and from other causes, both for the whole metropolis and the whole of each year, and for each parish and each week of the year may be learned.† Thinking that it would be interesting to contrast the ravages of plague in the seventeenth century with those of cholera in the nineteenth, I have been at some pains to arrange the facts in such a manner as may serve at the same time to convey some idea of the truly formidable character of the plague, and of the immensely improved condition of the public health in the present as compared with the earlier period. I have selected the three visitations of 1625, 1636, and 1665 as the subjects of investigation, because tolerably reliable calculations of the population of London have been made for periods sufficiently near to each of these visitations to enable the death-rates to be calculated with at least approximative accuracy.

* Loc. cit., pp. 247-53.

† Natural and Political Observations on the Bills of Mortality. By Capt. John Graunt, F.R.S. 1676.

London's Remembrancer; or a true Account of every particular week's Christenings and Mortality in all the years of Pestilence within the cognisance of the Bills of Mortality, being xviii years; taken out of the Register of the Company of Parish Clerks of London. By John Bell, Clerk to the said Company. 1665.

In 1631, a time of scarcity and threatened dearth, the Privy Council, in a letter still extant, applied to the Lord Mayor for a return of the number of mouths—men, women, and children—residing within the jurisdiction of the City. In the return—a copy of which is, I believe, still preserved at Guildhall—the population of each ward within and without the walls and in the borough of Southwark is specified. The total number of inhabitants within the city and its liberties, including Southwark, was 130,280. This was exclusive of Westminster, and of the several populous outlying parishes of Shoreditch, Whitechapel, Stepney, Bermondsey, etc., the population of which, if it could now be added to the return, would raise it to a number less disproportionate with the later computations. Captain Graunt estimated the population of London thirty years later, in the year 1661, to have amounted to 384,000, and King estimated it in 1685 at 530,000. The return of 1631 was intended for a return of the exact number residing within the jurisdiction of the Lord Mayor. The approximative correctness of the later computations is supported by the circumstance that the population of the same area, at the census of 1801, was 742,625. Supposing the earlier computation to have been accurate, the population of London only increased 40 per cent. during the 116 years that intervened between the time of Charles II. and that of the third George. Considering the great increase of commerce, the change of circumstances, and the additional inducements that existed to tempt persons to seek a residence in London towards the close of this period, it is probable that King's calculation exceeds, rather than falls below, the population of the metropolis at the period to which it refers.*

The average annual number of deaths returned in the bills of mortality for each of the six healthy years, 1631—1635 inclusive, was 9704. The total mortality returned for the years 1625 and 1636, when plague prevailed, was 54,265 for the former, and 23,359 for the latter year. The plague visitation of the first of these years, therefore, raised the mortality for that year to more than five times the average annual death loss. The visitation of 1636 was much less severe, and the loss of the metropolitan population by death during that year was less than two and a half times as great as the ordinary average of healthy years at that period. Fully to realise these facts, conceive the mortality of the cholera

---

* KING'S Natural and Political Observations. 1696. See, also, MACAULAY'S History of England, vol. i, p. 348, to which I am indebted for the information.

visitations of 1849 and 1854 raising the deaths in London from 68,755, the actual number in 1849, and 73,697, the actual number in 1854, to 266,885 for the earlier, and 132,442 for the later outbreak.* In 1625, no fewer than 35,417 fell victims to the pestilence. In 1636, 10,400 deaths were caused by plague.

Like cholera in our days, plague fell with unequal force upon different districts of the metropolis. The plague death-rate per 1000 persons within the city walls was 129 in 1625, and only 17 in 1636. The plague death-rate of the city without the walls, including the borough of Southwark, but excluding the suburban parishes, the population of which is unknown, was 289 in the 1000 in the more severe visitation of 1625, and 100 in the lighter visitation of 1636. The city mortality within the walls fell in the second outbreak to little more than an eighth; without the walls it exceeded a third, of that of the preceding visitation. A very analogous fact has been exhibited under our own observation. The cholera epidemic of 1853—probably in consequence of the vigorous sanitary inspection and improvements effected under the City Commissioners of Sewers—produced a mortality, less in comparison with that of 1849 than happened in most of the other metropolitan districts.

The differences of death-rate between smaller districts were even more remarkable. Portsoken ward lost 289 persons out of each 1000 by plague in the pestilence of 1625, and 128 in that of 1636. In Bishopsgate Without, the plague death-rates of the two periods were 183 and 132; Cripplegate Without, 362 and 135; Aldersgate Without, 170 and 69; Farringdon Without, 244 and 54; Southwark, 293 and 120. The general death-rates of these years were frightfully high. More than half the inhabitants of Bishopsgate and Cripplegate were numbered with the dead in 1625; and even in the city proper one-fifth of the population appears to have been swept away in the same fatal visitation. The general death-rates of 1636 varied from 56 in each 1000 inhabitants of the city proper, to 263 in Portsoken, and 317 in Bishopsgate Without.†

* The average mortality for the seven years 1845, 6, 7, 8, 50, 1, and 2, was 53,377.

† The deaths for each district have been extracted from the Bills of Mortality; the district populations used as divisors in calculating the death-rates are those returned to the Privy Council in 1631, the particulars of which may be found in Capt. Graunt's work, and have been reprinted in the Report of the Census of 1851. It is commonly asserted by contemporary authorities, that any decrease of the population of London occasioned by pestilence, was

The pestilence of 1665 is reported to have carried off 68,596 persons. The total number of deaths returned in the bill of that calamitous year was 97,306. The best contemporary authorities unanimously agree in believing the actual number of the dead to have considerably exceeded the official returns. The plague death-rate of that year, if Captain Graunt's calculation of the population was correct, must have been 178 in the thousand, and the general death-rate from all causes 253. If this be true, one-fourth of the population was carried away in a few months. But so great was the panic, that all who could do so, fled from the plague-stricken city, and the death-rates in the diminished population must have been higher than is indicated by even these formidable numbers. In the nine weeks commencing August 8th and ending October 10th, 60,000 deaths occurred. The heaviest mortality was in the week ending September 19th, or, according to the reformed calendar, September 8th. Upwards of 8,000 persons died in that week, and of these 7,165 are recorded to have succumbed to the epidemic. Three thousand persons died in one night; probably the most fatal night that ever occurred in London

No data exist from which to calculate the district death-rates of that period. Some idea of the mortality may be formed from employing the population of certain districts in 1801 as the divisor. Assuming, then, the population to have been the same in these districts in 1665 as in 1801, the plague of the former year was fatal to 112 out of each 1000 in St. Andrew's, Holborn; to 181 in St. Bride's; to 140 in St. Botolph's, Aldersgate; to 466 in St. Botolph's, Aldersgate Without; to 126 in St. Giles's, Cripplegate; to 231 in St. Sepulchre; and to 120 in the borough of Southwark. It is certain that in several of these districts the population was much greater in 1801 than in 1665. It is just possible that in others it may have diminished. I have, however, endeavoured to select districts in which there has probably been the least amount of variation. The highest metropolitan district cholera death-rate—that of Rotherhithe in 1849—was only 20·5 in the 1000. The cholera death-rate

---

always rapidly aud completely supplied by immigration. If the loss of population caused by the visitation of 1625 had not been entirely supplied when the city census of 1631 was taken, the death-rates here mentioned are excessive, just in proportion as the population of 1631 was less than that of 1625 anterior to the visitation. Even if the death-rates be on this supposition reduced so much as a third, and there is no reason to believe they ought to be so much reduced, the remainders will appear almost incredible when placed in juxtaposition with the death-rates of the present day.

of the Borough in 1849 was under 17 in the 1000. In 1854, although Southwark was one of the most fatal districts, the cholera death-rate was about 13 in the 1000. Contrast these figures with the plague death-rate of 1665, when 120 persons at least died out of each 1000 of the inhabitants of the same district. The city mortality from cholera in 1832 was at the rate of 5 in the 1000. In 1849—excluding the Unions of East and West London—it was 3·8 in the 1000, and in 1854 only 1·2 in the 1000. The plague death-rates of the city—the same city in local position, but how different in appearance and salubrity—in the visitations of 1625 and 1636 were, as I have already said, 129 and 17 in the 1000 respectively.

After sustaining such frightful losses from plague, it is not surprising that the mortality occasioned by alvine flux, the pestilence which succeeded the plague, should appear to have excited so little attention from contemporary writers. I have already contrasted the gross mortality caused by this form of disease, in two years of the seventeenth century, with the gross mortality produced by the two last visitations of cholera. The disease was of annual occurrence for nearly half a century. It sometimes destroyed 4000 persons in a year, and for twenty-five years together upwards of 2000 annually. Let us now compare the ten years, 1681-90, when the mortality from alvine flux had already begun somewhat to decline, and the ten years, 1846-55, which comprise the two last visitations of cholera, and all the years of the nineteenth century in which diarrhœa has been most fatal, for the purpose of contrasting the alvine flux death-rate of the seventeenth and present centuries. The annual average death-rate from this class of diseases, in the ten years of the seventeenth century, was 477 in the 100,000 persons. In the nineteenth century, it has only been 257. In the eighteenth century, which enjoyed the singular immunity from pestilence already referred to, it was most insignificant. In the ten years 1746-55, for which we possess an approximative estimate of the population—the alvine flux death-rate was only 26 in the 100,000.

Influenza is the only epidemic among those to which reference has been made in this paper which has in our days retained the same essential character that it possessed at the time of the sweating sickness, the plague, and the cholera and dysentery of the seventeenth century. The mortality occasioned by it has varied very much in different visitations. In some epidemics the general mortality of the year has been largely increased by it. In others the effect has been almost inappreciable. The deaths in the week, from February 3rd to 10th, 1733, were raised by influenza to 1588, making it the most fatal week that had occurred during the previous sixty-eight years. If this was raised in proportion to the present population of the metropolis, the deaths of that single week would be represented by 6718. The last severe epidemic of influenza—that of 1847—was fatal to somewhat more than 5000 persons over and above the usual mortality.* The difference of epidemic death-rate between the two periods has been computed by the Registrar-General at 10 in each 1000 of the population; the increase of the ordinary death-rate during the period of the visitation having been 31 in the 1000 in 1733, as compared with 21 in the 1000 in 1847. This is in accordance with the condition of the public health at the two periods, and also with the result of practical observation. Influenza is not often fatal in the healthy, but falls heavily upon persons previously feeble or delicate, and particularly upon such as were subjects of chronic or of latent pulmonary disease. Thus it is most fatal to the ill-fed, ill-clad, shivering population of the poorer districts where the tubercular *dyscrasia* prevails; more fatal in the insalubrious parts of cities, not because it there meets with any peculiar condition favourable to its development, but with an enfeebled population unable to resist its onslaught.

I must now endeavour briefly to illustrate the suggestions as to the best mode of studying epidemic diseases, enunciated at the commencement of this paper, by the more prominent points in the history of the several pestilences that have just been passed in review. Of these, each has been preceded or accompanied by certain cosmical phenomena. Three out of the five visitations of sweating sickness were preceded by unusually wet seasons. No mention is made of extraordinary humidity in connection with the fourth visitation. The summer and autumn of 1551 are said to have been hot and moist, and the weather of the whole year of an unusual character. Plague was on several occasions coincident with seasonal peculiarities. Mr. Bell, clerk to the company of Parish Clerks of London, speaks, in his account of the plague, of the unseasonableness of 1665 as one cause of the year's calamity. "And I conceive," says he, "that the contagion of the air doth arise from the unseasonableness of the weather; for the weather hath been very seldom, since the

* Tenth Annual Report of the Registrar-General, pp. xxvii-xxx. The 5,000 deaths were spread over a period of six weeks.

beginning of the plague, suitable to the season of the year, but the air hath been close and obnumbulated, insomuch that the sun hath not had power to do its office, which is to exhale all fogs and malign vapours." The influence of seasons on the several forms of alvine flux scarcely needs to be referred to. The hot summer of 1846 was characterised by an amount of mortality from diarrhœa and analogous disease unprecedented since the epidemic period of the eighteenth century. The late unusually hot season has presented the same phenomenon. Each of the three visitations of cholera has been attended by dryness of weather, cloudiness of sky, haziness of atmosphere, and an unusual height of the barometer. The atmosphere has been either still, or its movement below the average, and the temperature in the later visitations high.

There is one feature that has been presented by cholera which has also been noticed in regard to former pestilences. It is one also about which there can be little mistake, whereas, even if data of the kind existed, any attempt to compare the temperature or the weight and electrical condition of the atmosphere during the prevalence of the remoter pestilences, with the same phenomena in cholera years, would fail from the want of accurate means of observation in former periods. The feature to which I refer is the frequent prevalence of mist during periods of epidemic visitation. The last invasion of sweating sickness was inaugurated by impenetrable fogs of bad odour arising from the banks of the Severn. "These poisonous clouds of mist were observed moving from place to place, with the disease in their train," so that "whithersoever the winds wafted the stinking mist, the inhabitants became infected with the sweating sickness."* John Bell's observation on the fogs and malign vapours, which accompanied the last great outbreak of plague in London, has already been quoted. A non-professional, but highly competent observer in the north of England, directed attention to the existence of a sort of cloud, to which he gave the name of cholera cloud, during the visitations both of 1831-2 and 1848-9. The sky was overcast by a sort of misty screen during the calamitous visitation of cholera to Newcastle and Gateshead in 1853. Mr. Glaisher also, in his report to the General Board of Health, speaks of "the three epidemics of cholera as having been attended by a particular state of atmosphere, characterised by a prevalent mist,

---

* HECKER's Epidemics of the Middle Ages, pp. 291-2.

thin in high places, dense in low." Visitations of influenza have likewise been often accompanied by a prevalence of mist. The epidemics both of 1837 and 1847 were accompanied by an unusual haziness of the atmosphere in the north of England. The influenza visitation of 1847 was, in the metropolis, preceded by mist and gloominess. There was a remarkable darkness on Tuesday, November 16th. On the evening of Saturday, November 20th, "a dense fog lay over the Thames and London for the space of five hours." "On Sunday the sky was overcast."*

Probably the unusual fecundity, or at least the appearance of unusual swarms, of the lower animals, and the prolific production of microscopic fungi, which have so often accompanied epidemic pestilences, are at once the consequence and a proof of some unusual meteorological phenomena. Remarkable flights of locusts have frequently occurred in years of pestilence. Great numbers of flies are recorded to have appeared in 1664, the year in which the last great plague had its beginning. An insect, popularly known as the cholera fly, swarmed in the air both in the years of the earlier epidemics and at the time of the outbreak in Newcastle-on-Tyne in 1853. The occasional prevalence of murrain at the time of human pestilences, is also probably due to the lower animals being acted upon injuriously by the same general causes which so fatally affect the human race.

The apparently sudden outbreak of epidemic diseases is one of their commonest attributes in popular estimation, and has always been employed as an argument in favour of their importation. This suddenness is, however, only apparent; for, with few exceptions, epidemic outbreaks have been preceded by sufficient indications of the existence of an epidemic constitution tending towards, and more or less gradually passing into, the form of the approaching disease. The plague of 1665 was preceded, according to Sydenham, by a severe and unusual form of epidemic fever, into which it again subsided at its termination. It is indeed remarkable how the mortality from fever, and especially spotted fever, increased and waned along with plague. Just as in our own times, an unusual prevalence of diarrhœal flux has often preceded cholera, so was the cholera, so accurately described by Willis, preceded by diarrhœa.

The deaths recorded from diseases of the alvine flux character began to increase slightly several years anterior to the outbreak of cholera in 1831-32. The decline of analo-

* Registrar-General's Tenth Annual Report, p. xxvii.

gous diseases had been very gradual after the epidemic period of the seventeenth century. The annual number of deaths recorded in the bills of mortality, as caused by this form of disease, fell gradually from 1070 at the beginning, to but 20 at the close of the eighteenth century. The average number of deaths remained about 20 until 1823, in which year 48 deaths are recorded from this class of diseases. They again fell until 1827, when the number was 40, and in the four subsequent years 53, 41, 53 and 92. These numbers must not, indeed, be received as conveying an absolutely accurate impression of the mortality caused by alvine flux in these years. The bills of mortality were becoming more and more imperfect from year to year. They were probably relatively correct, and afford, if a faint, yet a true indication of some gradual change either in the *crasis* of the population, or in the circumstances by which it was encompassed, which, if it did not eventually produce, yet gave a predisposition for the approaching pestilence. It is worthy of note, that 48 of the deaths in 1831 were returned under the name of *cholera morbus.* Cholera in the pestilential form did not appear in any part of the metropolis until February 1832. Surely this fact, considered in conjunction with the numerous cases recorded in other parts of the country, many of which, if they were not identical with epidemic cholera, very closely resembled it, is a satisfactory proof that the magazine was ready prepared here, even if the spark which exploded it was brought from abroad.

Even influenza, which is the most sudden of epidemics, often appearing to attack almost a whole population in a few days, is sometimes preceded by diseases which gradually merge into the epidemic. Competent observers spoke of having observed cases of the same character some weeks prior to the great outbreak of January 1837; and Dr. Peacock, in his interesting account of the influenza of 1847, mentions the prevalence of pulmonary affections, attended by an uncommon amount of febrile disturbance, as having preceded the regular epidemic.

As neither plague nor cholera have sprung all at once into full activity, but have usually been preceded by milder diseases of an analogous character, so likewise both these diseases, and probably sweating sickness also, have perhaps been only malignant forms of diseases constantly more or less prevalent in London. It is usually said that plague visited London at intervals in a cyclical manner, the intervening years being presumed to have been healthy. Thus the five great outbreaks of 1593, 1603, 1625, 1636, and 1665 are frequently referred to. But scarcely a year passed over for nearly a century in which some deaths from plague are not recorded in the bills of mortality. The deaths from plague in the epidemic year 1603, amounted to upwards of 30,000; in 1604, 900 persons died of plague; in 1605, 400; in 1606, 2,000; in 1607, 2,000; in 1608, 2,000; in 1609, 4,000; in 1610, 1,800; from 1640 to 1648, upwards of 1,000 annually. In the years 1646 and 1647, the deaths amounted to 2,436 and 3,597 respectively. Maitland says, in his history of London, that for twenty-five years anterior to the great fire, the city had never been free from plague; and an examination of the bills of mortality shows that between 1603 and 1670, only three years occurred in which no deaths from plague were recorded.

Fever, certain forms of which appear to have held the same relation to plague which diarrhœa holds to cholera, produced, even in healthy seasons, nearly twice as many deaths in proportion to the population of London in the seventeenth century as in the present. The annual mortality from fever of all kinds, for several years anterior to the plague of 1665, exceeded 2,000. In 1661, including 335 cases of "spotted fever and purples," which were considered by contemporary writers as closely akin to the plague, the deaths from fever were 3,825. For eight years after the plague, the total annual mortality from fevers of all kinds never reached 2,000, and the average was under 1,500. In 1678 the deaths from fever, which for several years had, on the average, exceeded 2,000, rose to nearly 2,500; in 1679, to 2,800; in 1680, to 3,500. The mortality did not again retire within the limits of the eight healthy years, 1666-73, during the remainder of the century; but it gradually fell after 1680 until it reached 2,400 in 1683. It now again took a spring, and annually augmented until 1686, when 4,500 deaths were recorded. The deaths from fever henceforward exceeded 3,000 annually until 1694, when 423 deaths are recorded from spotted fever,—a disease already said to have been considered as a form of plague; and, including these, the mortality from fever amounted to 5,459. Fever was prevalent all the year; but it reached its climax in the autumn, and was most fatal in October. If the same rate of mortality were to occur in London for any single year at the present day, the deaths from fever would, in that year, amount to nearly 25,000. These epidemics of fever therefore, although less fatal than plague, bore a resemblance to it in their history, coming as they did in cycles, and dependant, as they must have been, upon the epidemic constitution of the time. Perhaps they were in their essential nature identical with plague, but some conditions necessary for the full development of the pestilence were absent. It was not unusual, long after the cessation of plague, for fever to be accompanied by phenomena which, in times of pestilential visitation, would have been considered characteristic of plague. Sir John Pringle, and Drs. Freind, Lind, and Munro, all describe fevers in jails, or among the troops, or the seamen of the fleet, which were accompanied by parotid abscesses, buboes, and carbuncles.*

The first cholera epidemic was, as has been above related, preceded by a gradual increase of analogous diseases. These diseases have ever since maintained an important position in the mortality register of London. The mortality they produced gradually increased from 1840 to 1845, when upwards of 2,500 deaths were caused by alvine flux, including diarrhœa, dysentery, and cholera under that head. From that time, the annual number of deaths from this cause, exclusive of years in which cholera has been epidemic, have never fallen so low as 2,000. Just as London was never free from plague during the period when plague was the great pestilence, so has it never been a single year free from cholera since the return of pestilence during the present century in that form of alvine flux. The fever of former days probably bore a typical resemblance, in respect of its relation to the pestilence of the period, with the diarrhœa of the present time. Pursuing, then, this analogy, the scattered cases of fatal cholera that annually happen—which, if slower in their course and upon the whole less malignant in type, are in character identical with epidemic cholera—are the representatives of the similarly scattered cases of plague which occurred in the healthier years of the plague period. During the plague period, there were some years in which the deaths from this pestilence were of insignificant amount. For seven or eight years anterior to 1665, the deaths from plague returned in the bills varied from 6 to 36; those from spotted fever from 114 to 368 in the year. Excluding the years 1848, 9, and 53, in each of which cholera was epidemic, the annual deaths from cholera during the years 1840-53 have varied from 28

* Pringle, Diseases of the Army, London, 1764, pp. 305, 328, 329. See also Observations on the Increase and Decrease of different Diseases, and particularly of the Plague, by William Heberden, jun., M.D., F.R.S., 4to., London, 1801, pp. 91-3.

to 228. In not fewer than six of the ten years they have exceeded 100. In each of the years 1846 and 1851 they exceeded 200. As fatal cases of plague formerly occurred which were not referable to any exotic source, so have there been many partial outbreaks of cholera of late years which have seemed to be of indigenous origin. The recent outbreak in West Ham appears to have been of this character.

Plague and cholera have further agreed in this respect, that the early cases of an epidemic outbreak have usually been the most malignant; the most rapid in their course; the most fatal in their termination. Each has also been considered as an imported disease, although it must be admitted that the views respecting the importation of plague entertained by the physicians of the seventeenth century were much more rational than those often maintained in regard to cholera at the present day. On this point, however, I must guard myself from misapprehension. I do not deny the importation of cholera any more than I deny the contagiousness of plague. The question must still be viewed as an unsettled one, requiring much careful investigation. The cause of pestilential cholera is probably to be found in the product of some particular form of the retrogressive transformation of alvine excretion. This decomposition may possibly take place spontaneously under definite conditions of temperature, moisture, and electricity. It may perhaps take place more readily in cholera excretions than in healthy ones; or the former may serve as a leaven to excite the peculiar putrefaction in the latter. It may be set up by a leaven capable of transmission by human intercourse in some manner as yet unknown. It may perhaps take place either in the moist accumulations of a cesspool or a sewer, or in the water of a river or a pool, provided this be of a certain temperature and sufficiently charged with the necessary organic factor. In the one case, the product of the decomposition will be inhaled in the form of miasm with the air; in the other, it may be drunk dissolved in the water. These points require careful, candid, honest investigation, and they are here referred to lest my previous narration of facts should be construed into an attempt to prove the exclusively indigenous origin of pestilence, and particularly of cholera. I cannot hesitate to believe in its indigenous origin; I dare not, in the present state of our knowledge, as an humble inquirer of nature, deny the possibility of its transmission from abroad. Plague probably was also due to some form of organic decomposition, but the subject of the process was of a different nature from that which produces cholera. However generated, the poison of plague or of cholera may require a peculiar *crasis* of the human subject; and this *crasis*, depending upon conditions participated in by large numbers of the population, may form a necessary predisposition for the disease. But I profess not to solve questions which can only be set at rest by much patient research and observation.

The chief conclusions that appear to be deducible from the facts here put together are, that of the several forms of epidemic diseases which have at different periods visited London, influenza has maintained its precise character from century to century, because it is independent of the extrinsic conditions of the population it attacks; black death, sweating sickness, plague, and epidemic cholera—whether in the seventeenth or nineteenth centuries—on the other hand, have appeared in succession and then disappeared, because they have required for their development some definite but temporary condition, either of their human subjects or of their places of abode. If a return to the ruder civilisation and habits of the fourteenth century were possible, London might again become liable to black death, which exists even in our own day in some remote provinces of Asia under the name of the *Pali* plague.* A return to the habits and diet of the sixteenth century might reproduce the personal *crasis*, which would lead to the outbreak of malignant rheumatic fever in the form called sweating sickness, which, however, was not altogether uninfluenced by surrounding circumstances. "Noxious exhalations from dung-pits, stagnant waters, swamps, impure canals, and the odour of foul rushes, which were in general use in the dwellings in England, together with all kinds of offensive rubbish, seemed not a little to contribute to it; and it was remarked universally, that wherever such offensive odours prevailed, the sweating sickness appeared more malignant."† The Roettingen sweating sickness of the early part of the present century, which coincided exactly in character with the sweating sickness of the fifteenth and sixteenth centuries, seems to attest the possibility of such an event.‡ A return to the dirty habits and unwholesome customs of our ancestors would almost certainly lead to the recurrence of plague, which still exists in places that in these respects bear some analogy, however faint, to London before

* For this fact I am indebted to a very interesting paper, written for the Epidemiological Society of London, by Dr. Hirsch, of Dantzic, and read at one of the meetings several years since.

† HECKER's Epidemics of the Middle Ages, p. 292. ‡ *Ibid.*, p. 325.

the great fire of 1666. The condition of London at that period may be learned from the writers of the day. The houses were encumbered with dirt and organic *débris* of the most revolting kind, but slightly screened from view by an occasional sprinkling of fresh rushes. The narrow and tortuous streets, undrained, unpaved, uncleansed, were the usual receptacle for all house and trade refuse, including the bodies of domestic animals, and often the offal of slaughtered cattle. Overhung by the projecting upper stories of houses, their ventilation was impeded by the cross signs then suspended from the premises of all tradesmen, a necessary token of residence in times when the numbering of houses was unknown. Cholera, perhaps caused in the seventeenth century by the want of the careful removal of excrement, has probably returned in our own century in consequence of the rapid growth of towns and the difficulty of removing their excrementitious products.

There remain some points of interest in the history of London pestilences to which I would like to have adverted. I could wish to have dwelt at greater length upon others to which I have referred more briefly than they deserved. Whether you adopt or reject the inferences I have deduced from the facts placed before you, I think we shall all agree in the importance of studying epidemic diseases in the broadest and most comprehensive manner. Excluding influenza, for the reason already assigned, it appears to me impossible to study with care and impartiality the history of the several pestilences which have been under consideration without recognising the necessity of certain home-bred elements for their production. They have in turn appeared, and they have in succession departed from London, not because they have at the one time been accidentally imported in bales of cotton, or by means of the sick, or at the other time effectually excluded by repressive precautions; but because at the one period the organic matter out of which plague or cholera is brewed abounded, and at the other period has been wanting. The exciter may perhaps have sometimes come to us over sea, but it would have failed to light up the epidemic conflagration had it not here met with materials upon which it could react.

